# The rise of the statins and the burdening inefficiencies in liver monitoring

**DOI:** 10.3399/bjgpopen20X101066

**Published:** 2020-04-01

**Authors:** Hashim Al-Gailani, Puneet Chadha

**Affiliations:** 1 GP, Stockport CCG, Stockport, UK; 2 GP, Birmingham & Solihull CCG, Birmingham, UK

**Keywords:** cardiovascular disease, guidelines, family medicine, primary healthcare

The prescribing of statins in the NHS has risen significantly over the years. In England, prescription items for statins that were dispensed in 2017 had increased by approximately 26 million compared to a decade ago.^1^ The updated National Institute for Health and Care Excellence (NICE) guidance in 2014, which opened up statins to those with cardiovascular risk of 10–20%, is a likely contributing factor to the increase in the later part of the last decade. More recently, NHS England’s improvement review sets out a plan, part of the wider ‘NHS long term plan’, which aims to improve identification and management of raised cholesterol as well as expanding access to genetic testing for familial hypercholesterolaemia (FH). More specifically, their vision includes statins becoming available through high street chemists. Thus, there is mounting evidence to suggest that statin prescribing is likely to increase further in the future, with an associated increase in liver monitoring.

Current NICE guidelines on cardiovascular disease recommends measurement of liver transaminase enzymes before starting a statin, within 3 months and at 12 months, but not again unless clinically indicated. An increase of alanine transaminase (ALT) greater or equal to three times the upper limit of normal (ULN) is associated with a greater risk of significant liver injury.^2^ Nevertheless, a study has shown that only 1% of participants had ‘clinically meaningful’ elevations in hepatic transaminases; that is, meeting threshold of 3 times upper limit of normal (ULN).^3^ This has been reflected in the updated NICE guidance with less frequent liver monitoring requirements.

Despite all the emphasis being on transaminases, most GPs in the NHS currently cannot request ALT testing alone. Instead, routine practice is to tick liver function tests (LFT) from the list when monitoring statins. A study has put this to the test and established a reduction of full LFT testing for those on statins by 24.3% if practitioners had access to ALT testing alone.^4^ This is a noteworthy result with huge cost implications when considering the huge rise in statin prescribing over the past decade. Furthermore, it will become more valuable when we take into account the NHS long term plan to improve outcomes in identifying and managing high cholesterol as well as FH.

In conclusion, we have highlighted the significant rise in statin prescribing in the past decade as well as the likely continued rise when considering updated NICE guidance which lowered the threshold and the NHS long term plan. It therefore becomes more important to be cost-effective when performing liver monitoring for these additional patients, while still meeting current guidance. It is also important to acknowledge the wider benefit to the patient in reducing the anxiety of additional blood tests.
